# Expression and prognostic value of transcription-associated cyclin-dependent kinases in human breast cancer

**DOI:** 10.18632/aging.202595

**Published:** 2021-03-03

**Authors:** Ning Li, Shaoquan Zheng, Zhicheng Xue, Zhenchong Xiong, Yutian Zou, Yuhui Tang, Wei-Dong Wei, Lu Yang

**Affiliations:** 1 Department of Breast Oncology, Sun Yat-Sen University Cancer Center, State Key Laboratory of Oncology in South China, Collaborative Innovation Center for Cancer Medicine, Guangzhou, China; 2 Department of Gastric Surgery, Sun Yat-Sen University Cancer Center, State Key Laboratory of Oncology in South China, Collaborative Innovation Center for Cancer Medicine, Guangzhou, China

**Keywords:** CDK, breast cancer, transcription, bioinformatics analysis, biomarker

## Abstract

The expression and prognostic significance of transcription-associated cyclin-dependent kinases (TA-CDKs) in breast cancer have not been systematically investigated. Using Oncomine, GEPIA2, the Human Protein Atlas, the Kaplan-Meier Plotter, cBioPortal, Metascape, and DAVID 6.8, we profiled the expression of TA-CDKs in breast cancer, inferred their biological functions, and assessed their effect on prognosis. The expression of CDK7/10/13/19 mRNAs in breast cancer tissues was significantly higher than in normal breast tissues. Survival analysis of breast cancer patients revealed that increased CDK8 expression was associated with inferior overall survival (OS), higher expression of CDK7 or CDK8 was associated with inferior relapse-free survival (RFS), but higher expression of CDK13 was associated with favorable RFS and OS. In addition, a high genetic alteration rate (56%) in TA-CDKs was associated with shorter OS. On functional enrichment analysis, top GO enrichment items for TA-CDKs and their neighboring genes included cyclin-dependent protein serine/threonine kinase activity and transferase complex. The top KEGG pathways included cell cycle and mismatch repair. These results suggest that CDK7/8/13 are potential prognostic biomarkers for breast cancer patients and provide novel insight for future studies examining their usefulness as therapeutic targets.

## INTRODUCTION

Breast cancer remains the most common malignancy and the leading cause of cancer mortality for women worldwide, accounting for 24.2% of total cancer cases and 15.0% of total cancer deaths [1]. Therapy concepts for breast cancer have taken locoregional tumor load and molecular subtype into account. Breast cancer is generally classified into four main molecular subtypes: luminal A, luminal B, human epidermal growth factor receptor 2 (HER2)-enriched, and triple-negative [2, 3]. Although therapy has progressed substantially over the past years—following adoption of multidisciplinary treatment including surgery, radiotherapy, chemotherapy, and endocrine and anti-HER2 therapies—, 20%-30% of patients develop distant metastasis and present poor prognosis [4]. This therefore highlights the need for identifying biomarkers with a crucial role in the development of breast cancer, to improve patient diagnosis and prognosis.

Cell cycle deregulation is a defining hallmark of cancer [5]. Differential expression and mutational changes in cyclin-dependent kinases (CDKs), a set of key regulatory enzymes that drive cell cycle transitions, have been shown to contribute to the development of numerous neoplasias [6]. Besides their classical function in cell cycle control, a more extensive role in the transcriptional regulation of gene expression has been revealed for several of the more than 20 members of the CDK family identified so far [6]. The CDK family is broadly divided into two subfamilies: cell cycle-associated CDKs (for example, CDK1, CDK2, CDK4, and CDK6) that directly promote cell cycle progression, and transcription-associated CDKs (TA-CDKs, i.e., CDK7/8/9/10/11/12/13/19/20) that regulate gene transcription [6, 7]. TA-CDKs are conserved both in sequence and function and regulate gene transcription by reversibly phosphorylating the carboxy-terminal domain of the largest subunit (Rpb1) of RNA polymerase II, a major but not exclusive, TA-CDK target [8]. Despite this categorization, the function of many family members of the TA-CDK class are frequently combined. Given that CDKs play crucial roles in cancer cell survival, many efforts have been made to exploit strategies to inhibit CDKs in cancer cells. This has led to the development of CDK4/6 inhibitors (palbociclib, ribociclib, and abemaciclib), which have been approved by the FDA for the treatment of estrogen receptor (ER)-positive breast cancer [9]. The addition of CDK4/6 inhibitors to endocrine therapy has resulted in a significant improvement in progression-free survival (PFS) for this subtype of breast cancer [10]. In contrast, although accumulating evidence suggests that TA-CDKs have important functions in cancer, their precise involvement in breast cancer remains inconclusive, and thus their potential as therapeutic targets has not been clearly established [8].

The expression patterns of TA-CDKs and their relationship with clinicopathological characteristics and prognosis have not been fully reported in breast cancer. With the rapid development of microarray and RNA-sequencing technologies and the establishment of various public databases, comprehensive analysis of TA-CDKs has become feasible. In this study, we conducted a comprehensive bioinformatics analysis of the expression and mutations of TA-CDKs in patients with breast cancer and assessed their potential value as prognostic biomarkers. Our results provide novel insights that may foster prognostic accuracy in breast cancer and highlight the relevance of TA-CDKs as attractive therapeutic targets to improve patient outcomes.

## RESULTS

### Aberrant expression of TA-CDKs in patients with breast cancer

**Figure 1:**
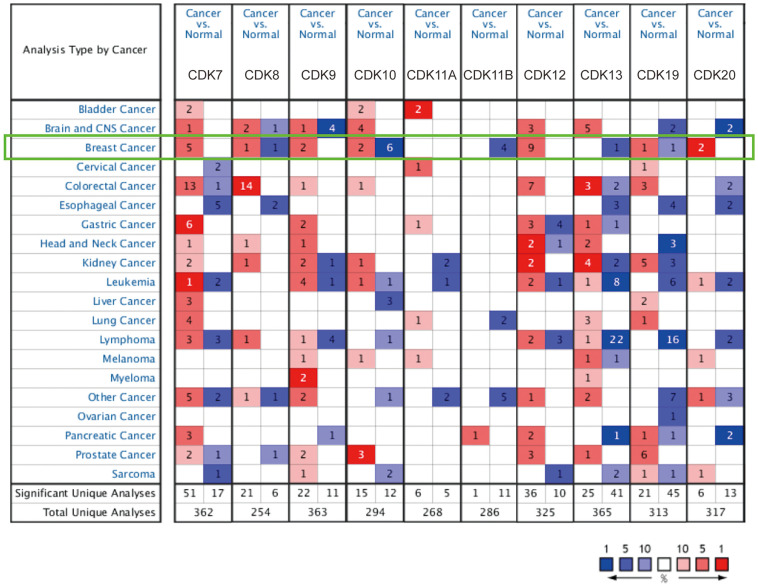
**TA-CDK mRNA expression levels in different types of cancers (ONCOMINE).** The numbers of datasets with overexpression (red) or underexpression (blue) of the target gene are shown.

We first performed comparative transcriptional analysis of 10 TA-CDKs using breast cancer and normal breast tissue data from the Oncomine database. The results revealed that the mRNA expression of CDK7, CDK8, CDK9, CDK10, CDK12, CDK19, and CDK20 was upregulated in patients with breast cancer (Figure 1 and Table 1). Further comparisons with normal breast tissue revealed that CDK7 was upregulated in ductal breast carcinoma (fold change = 1.734) in Sorlie’s dataset, and in lobular breast carcinoma (fold change = 1.841) and ductal breast carcinoma (fold change = 1.697) in Sorlie’s dataset 2. CDK7 mRNA was also elevated in lobular breast carcinoma (fold change = 1.761) and in ductal breast carcinoma (fold change = 1.991) in Zhao’s dataset. In turn, CDK8 mRNA expression was increased in ductal breast carcinoma (fold change = 1.670) in Richardson’s dataset. CDK9 was found to be highly expressed in invasive breast carcinoma (fold change = 1.725) in the TCGA dataset, and in invasive lobular breast carcinoma (fold change=1.778) in Turashvili’s dataset. Radvanyi et al. showed that CDK10 transcription levels were increased in invasive mixed breast carcinoma (fold change = 2.056) and in invasive lobular breast carcinoma (fold change = 1.590). Higher expression of CDK12 was found in both invasive ductal breast carcinoma (fold change = 3.586) and invasive mixed breast carcinoma (fold change = 3.193) in Radvanyi’s database, and in invasive ductal breast carcinoma (fold change=1.938) and invasive lobular breast carcinoma (fold change = 1.603) in Zhao’s dataset. Compared also to normal tissue, CDK12 was found to be overexpressed in invasive ductal and lobular carcinoma, invasive breast carcinoma, and intraductal cribriform breast adenocarcinoma in the TCGA dataset, in invasive breast carcinoma in Gluck’s dataset, and in ductal breast carcinoma *in situ* in Ma’s dataset. CDK19 was increased in invasive ductal breast carcinoma (fold change = 1.682) in TCGA. Higher CDK20 expression was in turn found in both intraductal cribriform breast adenocarcinoma (fold change = 2.225) and invasive ductal and lobular carcinoma (fold change = 1.645) in TCGA.

**Table 1:** Differential transcriptional expression of TA-CDKs in different types of breast cancer (Oncomine database).

**Gene**	**Breast cancer type**	**P value**	**T test**	**Fold change**	**Source and/or reference (PMID)**
CDK7	Lobular breast carcinoma vs. Normal	0.007	3.101	1.841	Sorlie Breast 2 (12829800)
	Ductal breast carcinoma vs. Normal	8.26E-4	4.773	1.697	Sorlie Breast 2 (12829800)
	Lobular breast carcinoma vs. Normal	8.45E-7	7.576	1.761	Zhao Breast (15034139)
	Invasive ductal breast carcinoma vs. Normal	2.05E-6	12.305	1.991	Zhao Breast (15034139)
	Ductal breast carcinoma vs. Normal	0.004	3.975	1.734	Sorlie Breast (11553815)
CDK8	Ductal breast carcinoma vs. Normal	6.93E-6	4.877	1.670	Richardson Breast 2 (16473279)
CDK9	Invasive ductal and lobular carcinoma vs. Normal	2.17E-4	6.172	1.725	TCGA Breast
	Invasive lobular breast carcinoma vs. Normal	0.028	2.130	1.778	Turashvili Breast (17389037)
CDK10	Invasive mixed breast carcinoma vs. Normal	0.005	3.224	2.056	Radvanyi Breast (16043716)
	Invasive lobular breast carcinoma vs. Normal	0.039	1.925	1.590	Radvanyi Breast (16043716)
CDK12	Invasive ductal breast carcinoma vs. Normal	0.002	3.794	3.586	Radvanyi Breast (16043716)
	Invasive mixed breast carcinoma vs. Normal	0.007	4.501	3.193	Radvanyi Breast (16043716)
	Invasive ductal breast carcinoma vs. Normal	3.51E-7	5.940	1.938	Zhao Breast (15034139)
	Lobular breast carcinoma vs. Normal	3.86E-4	3.930	1.603	Zhao Breast (15034139)
	Invasive ductal and lobular carcinoma vs. Normal	1.96E-25	12.876	1.799	TCGA Breast
	Invasive breast carcinoma vs. Normal	7.88E-14	8.244	1.678	TCGA Breast
	Intraductal cribriform breast adenocarcinoma vs. Normal	0.005	5.291	1.979	TCGA Breast
	Invasive breast carcinoma vs. Normal	7.33E-4	6.866	1.849	Gluck Breast (21373875)
	Ductal breast carcinoma *in situ* vs. Normal	0.001	3.956	1.588	Ma Breast 4 (19187537)
CDK19	Invasive ductal breast carcinoma vs. Normal	1.67E-28	14.135	1.682	TCGA Breast
CDK20	Intraductal cribriform breast adenocarcinoma vs. Normal	3.23E-19	17.058	2.225	TCGA Breast
	Invasive ductal and lobular carcinoma vs. Normal	2.04E-6	8.862	1.645	TCGA Breast

Normal: non-tumor breast tissue.

**Figure 2:**
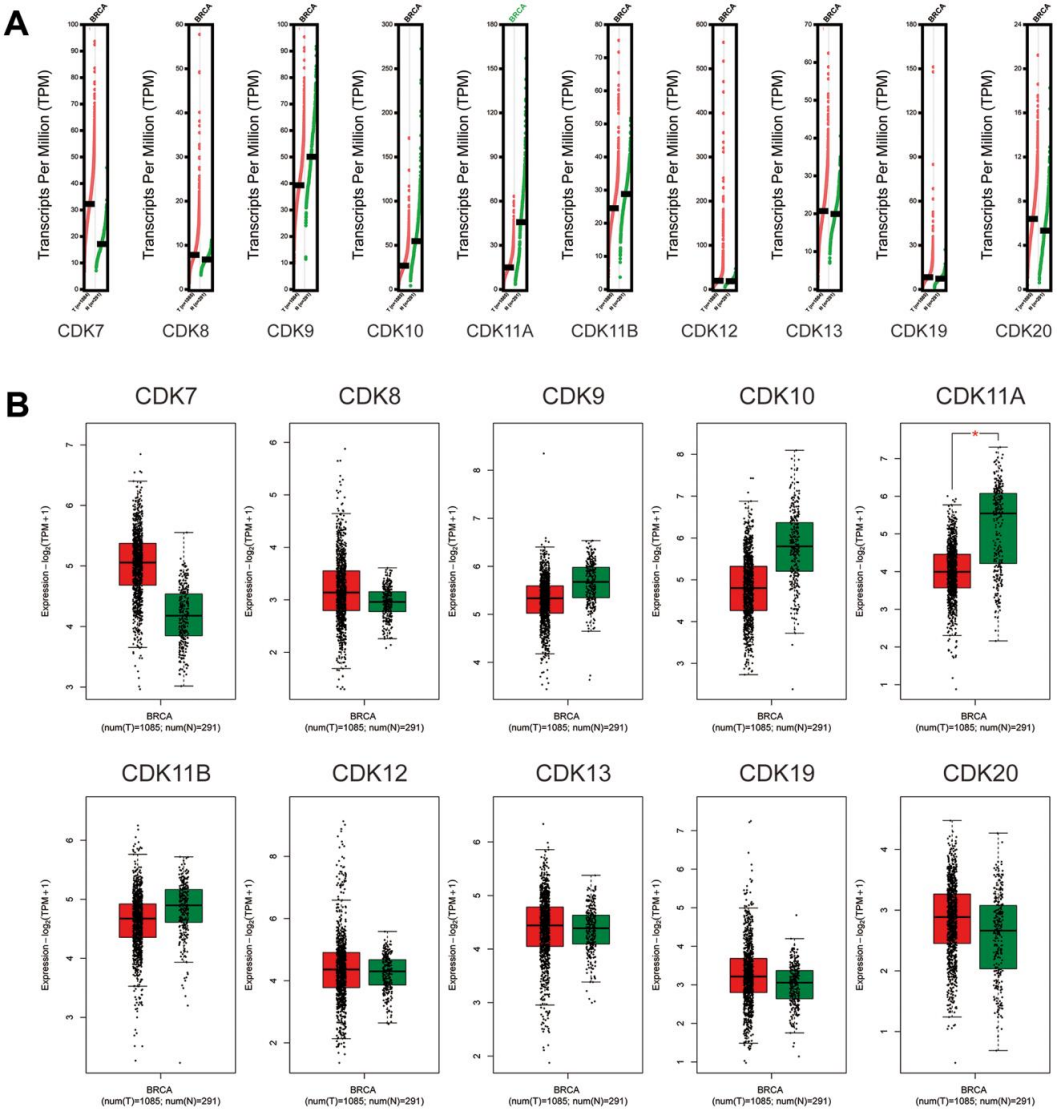
**Expression of TA-CDK mRNAs in breast cancer (GEPIA2).** (**A**) Scatter diagram. (**B**) Box plot. *P < 0.05.

Next, we further compared the transcriptional expression of the above CDKs in breast cancer and normal tissues in the GEPIA2 database. The results showed that although the mRNA expression of CDK7, CDK8, CDK19, and CDK20 was higher in breast cancer tissues, none of these transcripts’ values reached statistical significance. In contrast, CDK11A mRNA levels were significantly lower in breast cancer tissues than in normal ones (Figure 2). We also analyzed potential relationships between the mRNA levels of TA-CDKs and tumor stage in patients with breast cancer in GEPIA2. The results showed no correlation for any TA-CDK mRNA with patients’ cancer stages (Supplementary Figure 1). Meanwhile, on the TCGA portal, we found that CDK7, CDK8, CDK9, CDK12, CDK19, and CDK20 mRNA levels were differentially expressed in the 4 molecular tumor subtypes (Supplementary Figure 2). Expression and mutation analysis in the TCGA portal showed that PIK3CA, TP53, and CDH1 were the 3 most common mutated genes associated with dysregulated expression of the 10 TA-CDKs (Supplementary Figure 3).

**Figure 3:**
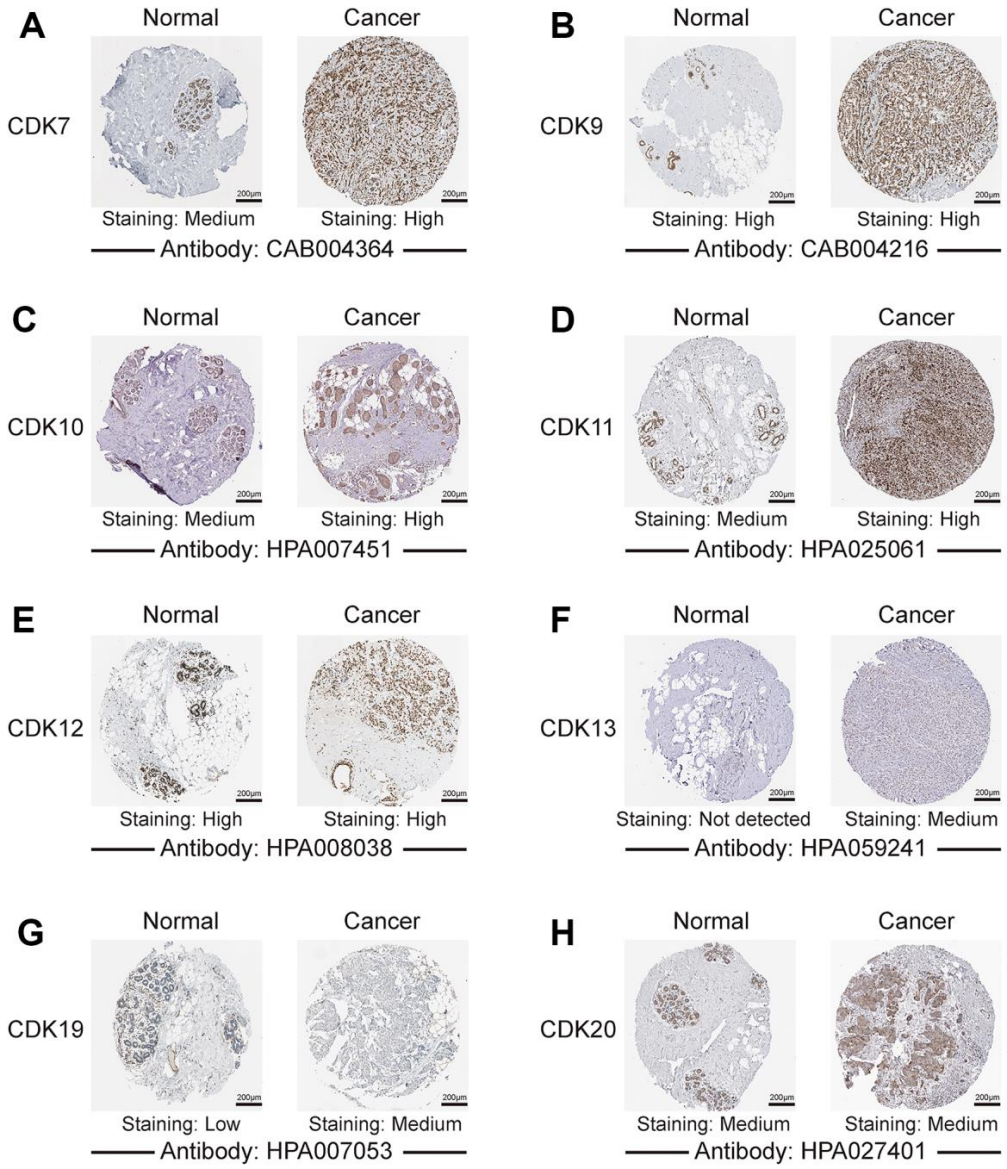
**Representative images of TA-CDK immunohistochemistry in normal breast and breast cancer tissues (Human Protein Atlas).** (**A**) CDK7. (**B**) CDK9. (**C**) CDK10. (**D**) CDK11. (**E**) CDK12. (**F**) CDK13. (**G**) CDK19. (**H**) CDK20.

We further explored the protein expression patterns of TA-CDKs in the Human Protein Atlas (HPA). CDK13 and CDK19 were not expressed or lowly expressed in normal breast tissues, whereas medium expression of these two proteins was observed in breast cancer tissues (Figure 3). CDK7, CDK10, and CDK11 were moderately expressed in normal breast tissues and highly expressed in breast cancer tissues. In turn, high expression of CDK9 and CDK12 and medium expression of CDK20 were observed in both tissue types (Figure 3). These results indicated that aberrant expression of several TA-CDKs also occurs at the protein level in patients with breast cancer.

### Prognostic value of the expression of TA-CDKs in patients with breast cancer

**Figure 4:**
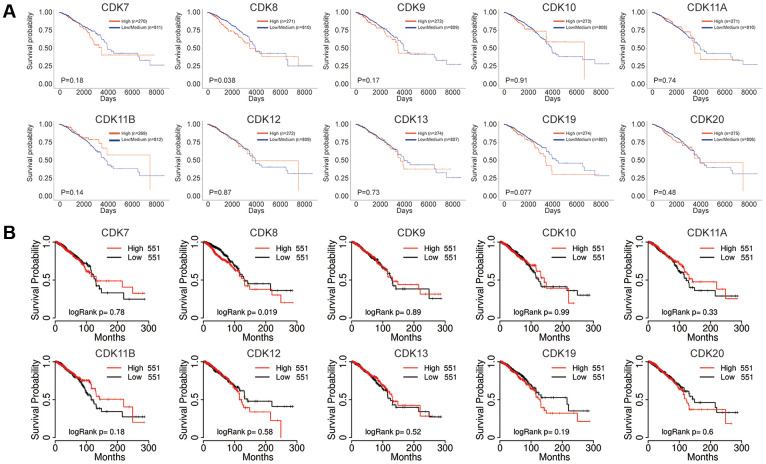
**Prognostic value of the mRNA expression of distinct TA-CDKs in breast cancer patients.** (**A**) UALCAN tool. (**B**) TCGA portal.

We next explored the prognostic value of TA-CDKs in patients with breast cancer using the UALCAN, TCGA portal, and Kaplan-Meier Plotter platforms. Increased CDK8 mRNA levels were associated with shorter OS in UALCAN (P = 0.038), TCGA portal (P = 0.019) and Kaplan-Meier Plotter (P = 0.015) (Figure 4 and Supplementary Figure 4). Higher mRNA expression of CDK19 tended to be associated with shorter OS in UALCAN (P = 0.077). In the Kaplan-Meier plotter database, higher combined expression of the CDK7, CDK8, CDK9, CDK10, CDK12, and CDK13 mRNAs was associated with poorer RFS (HR, 1.19, P = 0.03, Supplementary Figure 4A). On analyses of individual CDK transcripts, higher expression of CDK7 (HR, 1.21, P<0.001) and CDK8 (HR, 1.48, P<0.001) was associated with inferior RFS, whereas higher CDK13 levels were associated with favorable RFS (HR, 0.72, P<0.001) and increased OS (HR, 0.67, P = 0.012) (Supplementary Figure 4A, 4B). In contrast, the individual mRNA expression levels of CDK9, CDK10, CDK11A, CDK11B, CDK12, and CDK20 showed no significant correlation with prognosis in breast cancer patients. These results indicated that the transcriptional expression levels of CDK7/8/13/19 represent prognostic factors for breast cancer and might be exploited as biomarkers for prognosis evaluation and individualized therapy.

### Genetic mutations in TA-CDKs and associations with prognosis in patients with breast cancer

**Figure 5:**
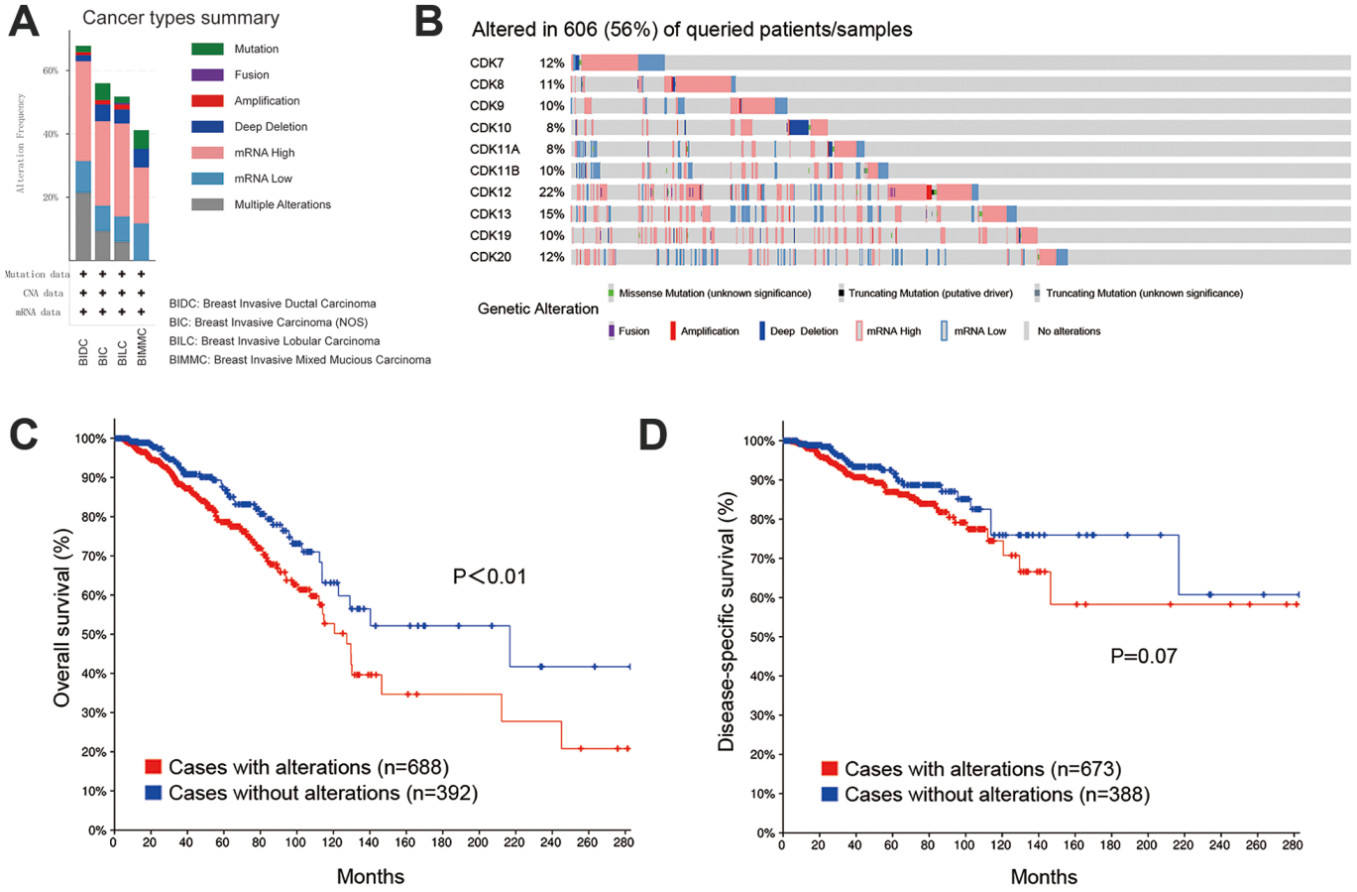
**Genetic alterations in TA-CDKs and their association with OS and DSS in breast cancer patients (cBioPortal).** (**A**) Summary of alterations in TA-CDKs in different breast cancer types. (**B**) OncoPrint visual summary of genetic alterations detected in TA-CDKs. (**C**) Kaplan–Meier curves comparing OS in breast cancer cases with or without genetic alterations in TA-CDKs. (**D**) Kaplan–Meier curves comparing DSS in breast cancer cases with or without genetic alterations in TA-CDKs.

We next accessed the cBioPortal tool to evaluate potential correlations between genetic alterations in TA-CDKs and prognosis in breast cancer. A total of 1082 patients on the TCGA PanCancer dataset were analyzed. Among the 4 different breast cancer types, the genetic alteration rate ranged from 29.4% to 60.9% (Figure 5A). The percentages of genetic alterations in TA-CDKs varied from 8 to 22% for individual genes, with an overall alteration rate of 56% (606/1082) in the queried patients (CDK7, 12%; CDK8, 11%; CDK9, 10%; CDK10, 8%; CDK11A, 8%; CDK11B, 10%; CDK12, 22%; CDK13, 15%; CDK19, 10%; CDK20, 12%; Figure 5B). Kaplan-Meier plots demonstrated that genetic alteration of these CDKs was significantly associated with inferior OS (P<0.01) (Figure 5C), and tended to confer also shorter disease-specific survival (DSS, P = 0.07) (Figure 5D). These results suggest that genetic alterations in TA-CDKs occur at a high rate in breast cancer patients and are associated with unfavorable prognosis.

### Functional enrichment analysis of TA-CDKs and neighboring genes in breast cancer

**Figure 6:**
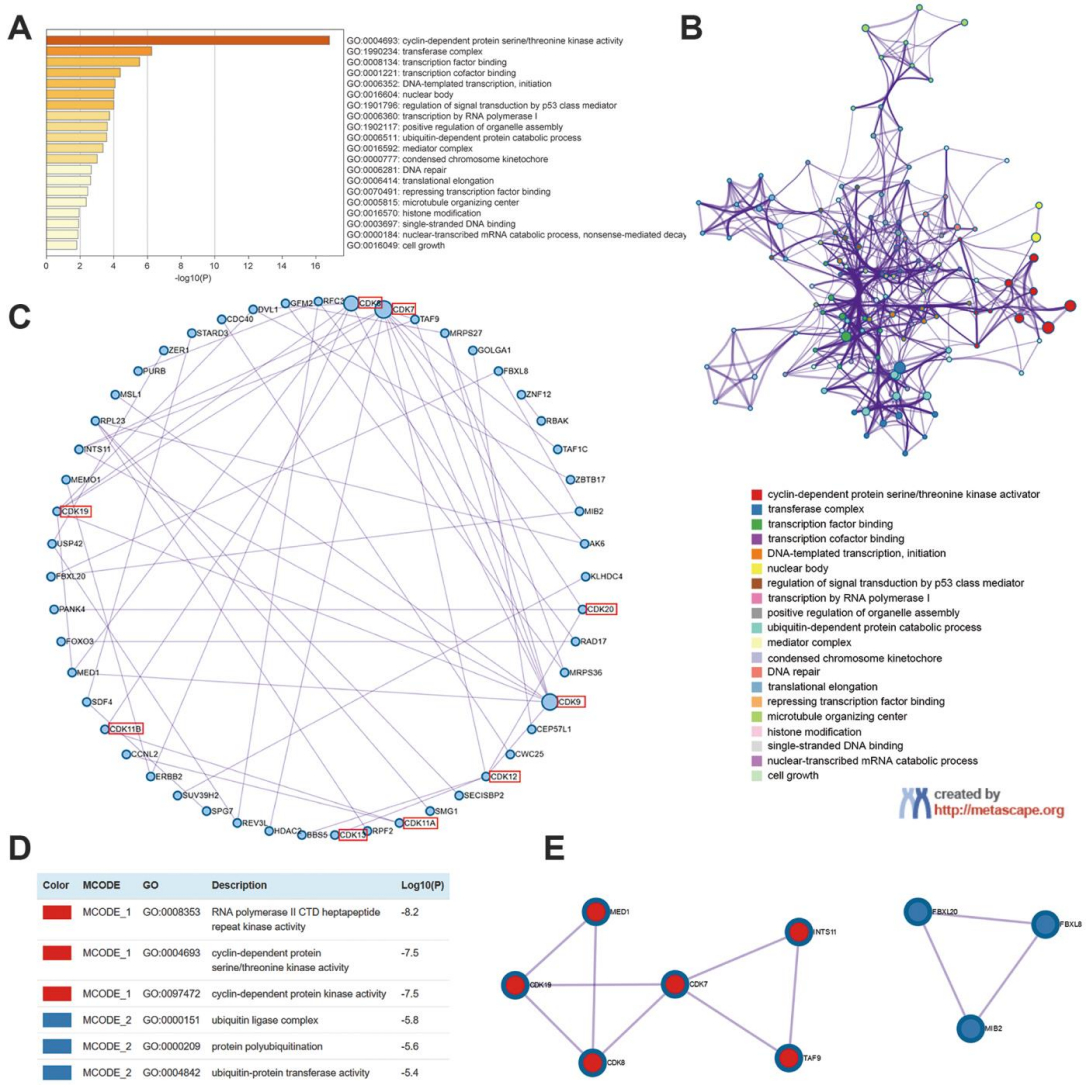
**Enrichment analysis of TA-CDKs and their closest 100 neighboring genes in breast cancer (Metascape).** (**A**) Heatmap of GO enriched terms colored by p-values. (**B**) Network of GO enriched terms colored by cluster. (**C**) Protein-protein interaction enrichment analysis (degree-sorted circular layout). (**D**) Functional enrichment analysis of the MCODE components. (**E**) Network representation of the major protein interaction clusters identified by the MCODE algorithm.

We next identified the 100 closest neighboring genes significantly associated with the expression of TA-CDKs in the GEPIA2 platform and conducted functional and pathway enrichment analyses of these gene sets in Metascape. The top 20 GO enrichment items included cyclin-dependent protein serine/threonine kinase activity, transferase complex, transcription factor binding, transcription cofactor binding, DNA-templated transcription, nuclear body, and regulation of signal transduction by p53 class mediator, among others (Figure 6A). The representative terms from GO analysis were then converted into a network (Figure 6B), and a protein-protein interaction enrichment analysis was subsequently performed (Figure 6C). Upon identification of MCODE components in the corresponding gene lists, biological function analysis indicated main relationships with RNA polymerase II CTD heptapeptide repeat kinase activity, cyclin-dependent protein serine/threonine kinase activity, cyclin-dependent protein kinase activity, ubiquitin ligase complex, protein polyubiquitination, and ubiquitin-protein transferase activity (Figure 6D, 6E).

**Figure 7:**
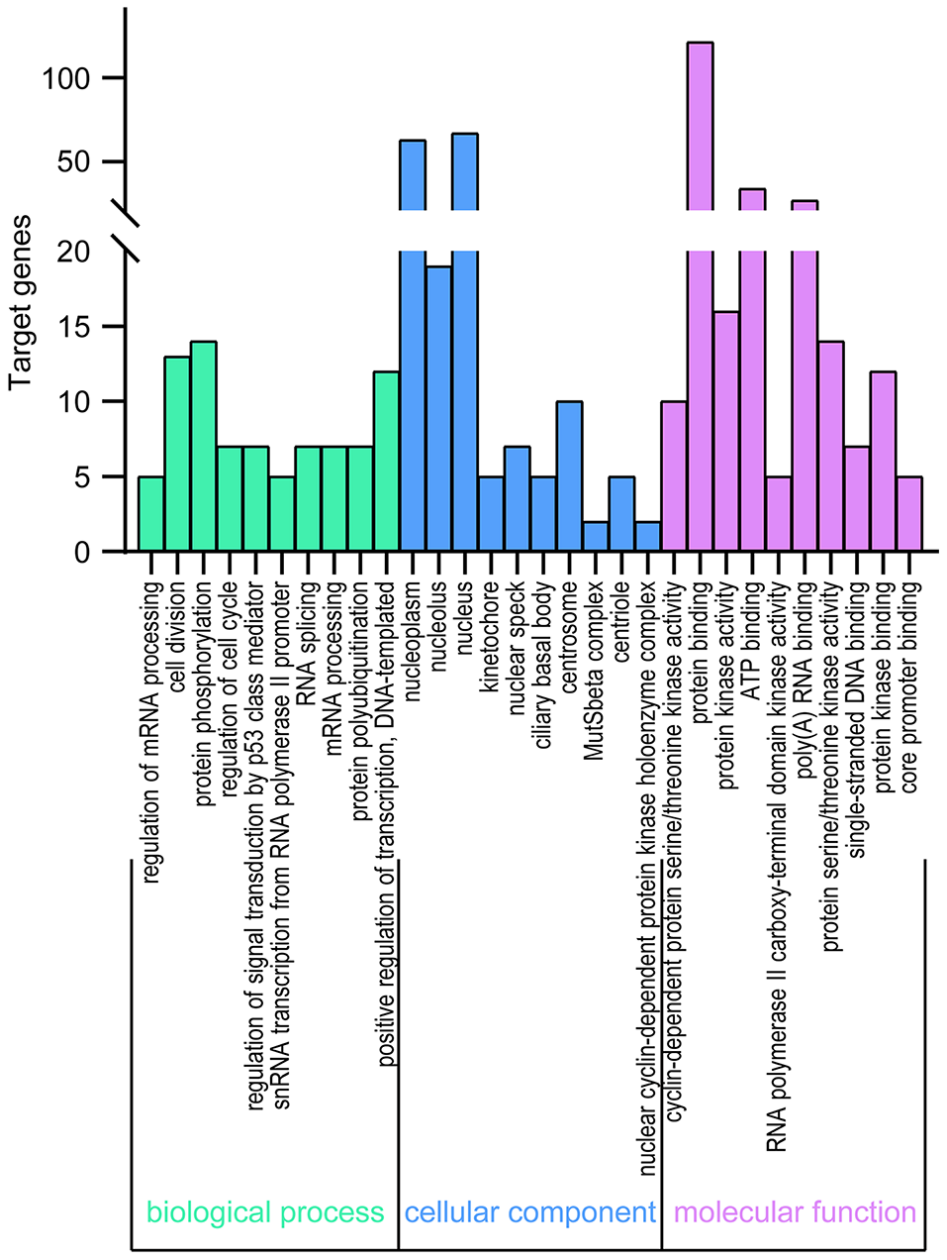
**GO enrichment of TA-CDKs and their closest 200 neighboring genes.** Significant GO terms across the CC, BP, and MF categories were extracted using DAVID 6.8.

**Figure 8:**
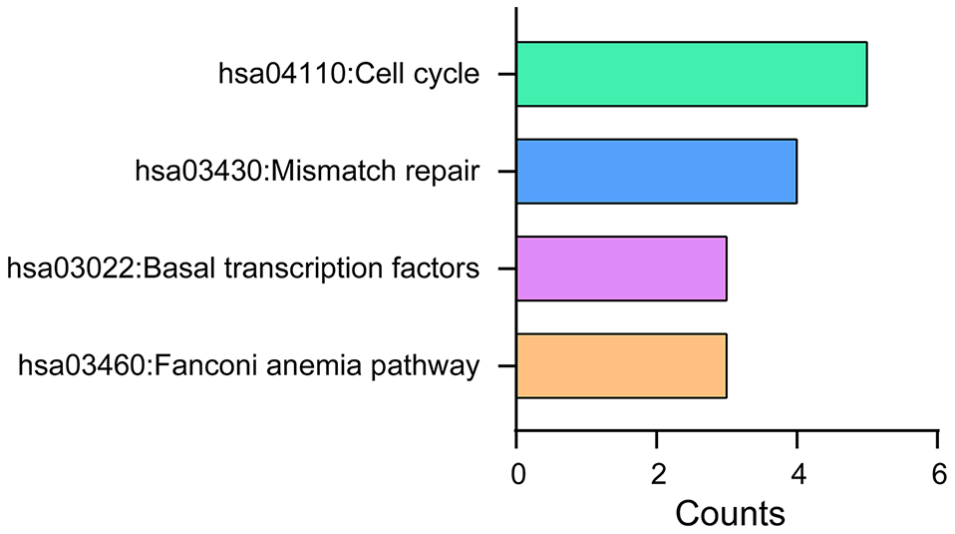
**KEGG pathway analysis of TA-CDKs and their closest 200 neighboring genes.** The analysis was performed using the DAVID 6.8 tool.

**Figure 9:**
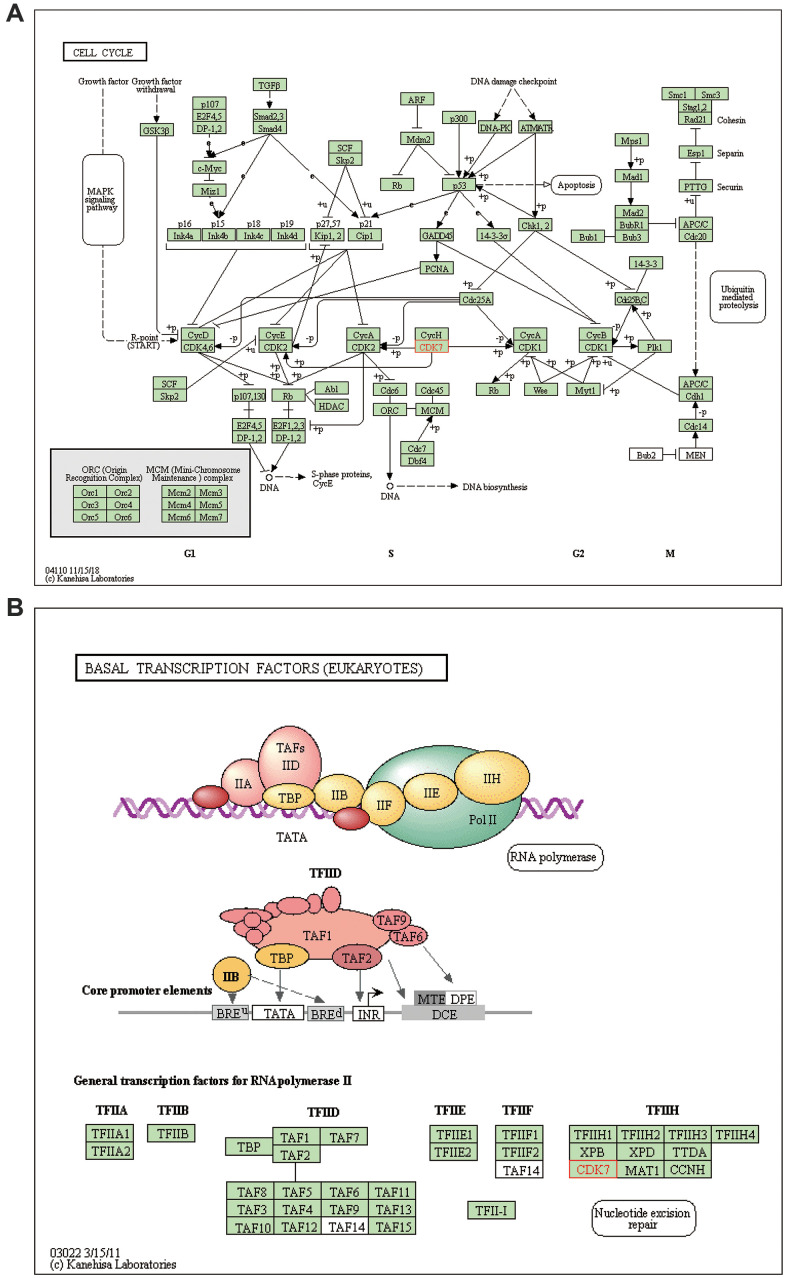
**Regulatory actions of TA-CDKs in breast cancer.** (**A**) Cell cycle processes/pathways. (**B**) Pathways involving basal transcription factors.

DAVID 6.8 was further utilized to predict the functions of TA-CDKs and their closest 200 neighboring genes through GO and KEGG analyses. The 10 most highly enriched functions in BP, CC, and MF are shown in Figure 7. As expected, the top KEGG pathways included hsa04110: Cell cycle, hsa03430: Mismatch repair, and hsa03022: Basal transcription factors (Figure 8), all of which are involved in the tumorigenesis and pathogenesis of breast cancer (Figure 9).

## DISCUSSION

Although the fundamental role of CDKs in cell cycle control has been firmly established, multiple recent studies provide mounting evidence for the involvement of these kinases in other functions, such as gene transcription, DNA damage repair, cell death, and differentiation. Stemming from these findings, TA-CDKs are emerging as critical tumor biomarkers and targets in cancer treatment [7]. Numerous studies have suggested that TA-CDKs are involved in regulating gene expression at multiple levels, affecting transcription, splicing, and epigenetic modifications [6, 8]. However, although TA-CDKs have been show to impact tumorigenesis and metastasis in several malignancies, their prognostic value and biological function in breast cancer remain to be fully elucidated. Therefore, in this study we systematically explored the expression patterns, genetic alterations, potential functions, and prognostic utility of TA-CDKs in breast cancer. Our results indicate that CDK7, CDK8, and CDK13 could be prognostic biomarkers for breast cancer patients.

CDK7 has a general role in the phosphorylation of the carboxyterminal domain of RNA polymerase II that contributes to the initiation of transcription [11]. The expression of CDK7 and its cofactors cyclin H and MAT1 was found to be elevated in breast cancer compared with normal breast tissue. Interestingly, survival analysis showed an association between CDK7 expression and better outcome [12]. In our study, analysis of Oncomine and HPA datasets revealed that CDK7 expression was significantly higher in breast cancer than in normal breast tissue. In turn, survival analysis indicated that higher mRNA expression of CDK7 was significantly associated with inferior RFS in breast cancer patients. CDK7 knockout can lead to the exhaustion of adult stem cells [13], and its inhibition was shown to enhance anti-tumor immunity in small-cell lung cancer (SCLC) [14]. Inhibition of CDK7 suppressed the metastasis of osteosarcoma [15]. The covalent CDK7 inhibitor THZ1 showed potent efficacy in human T-cell acute lymphoblastic leukemia, which is dependent on transcription for maintenance of the oncogenic state [16]. Subsequently, other transcriptionally addicted malignancies, including MYCN-amplified neuroblastoma and MYC/MYCL-amplified SCLC showed also sensitivity to CDK7 inhibition [17]. Triple-negative breast cancer (TNBC) cells are exceptionally dependent on CDK7, and a vital cluster of genes in TNBC is especially sensitive to CDK7 inhibition [18]. Accordingly, CDK7 inhibition induced apoptotic cell death and inhibited the growth of patient-derived xenografts of TNBC [18]. The above results suggest that CDK7 may represent a therapeutic target in breast cancer.

CDK8 is part of the Mediator complex that regulates transcription [11] and can function as either a tumor suppressor or an oncogene in different contexts. In colorectal cancer, CDK8 may function as an oncogene by regulating β-catenin activity [19]. CDK8 overexpression was detected in a subset of melanoma cells with macroH2A loss and suppression of CDK8 inhibited the proliferation of melanoma cells [20]. In contrast, CDK8 deletion in the Apc^Min^ intestinal tumor model led to shortened survival and increased tumor burden [21]. The tumor suppressor effect of CDK8 was also observed in endometrial cancer [22]. Inhibition of CDK8 with cortistatin A has an anti-leukemic effect both *in vitro* and *in vivo* [23]. Although CDK8 inhibition exhibited weak antiproliferative activity in colon cancer cell lines [24], the CDK8/19 inhibitor Senexin B exerted a potent antitumor effect and augmented the effects of fulvestrant on ER-positive breast cancer [25]. In our study, increased mRNA levels of CDK8 were significantly associated with inferior OS and RFS for patients with breast cancer in the TCGA portal and in the UALCAN and Kaplan-Meier Plotter databases. Along with available preclinical data, these findings therefore support a pro-oncogenic role for CDK8 in breast cancer.

CDK13 is a close homolog of CDK12, with their kinase domains sharing ~92% identity [7]. This similarity makes it difficult to generate specific CDK12 or CDK13 inhibitors. CDK12 function is associated with the expression of a restricted set of DNA damage response (DDR) genes. Studies have suggested that loss-of-function mutation of CDK12 may lead to sensitivity to PARP inhibitors, platinum chemotherapy, and other targeted agents [7]. Interestingly, inactivation of CDK12 could identify a subgroup of advanced prostate cancer that may benefit from immune checkpoint inhibitors [26]. Knockout of CDK12 in an *in vivo* osteosarcoma model of lung metastasis significantly decreased the ability of OS to metastasize the lung [27]. In patient-derived xenografts models from patients with heavily pre-treated ovarian cancer, THZ1 (a chemical that inhibits CDK7, CDK12, and CDK13) repressed MYC expression and suppressed tumor growth. Notably, MYC suppression required the combined inhibition of CDK7, CDK12, and CDK13 [28]. In our study, the expression levels of CDK13 in breast cancer tissues were significantly higher than in normal breast tissues. Survival analysis showed that although higher mRNA expression of CDK13 in breast cancer was significantly associated with favorable RFS and OS in the Kaplan-Meier Plotter, genetic alteration in transcription-associated CDKs was instead associated with shorter OS in cBioPortal. In HER2-enriched breast cancer, CDK12 promotes tumor initiation and trastuzumab resistance, while CDK12 inhibition enhances the efficacy of trastuzumab [29]. In TNBC, the novel CDK12/CDK13 inhibitor SR-4835 has been shown to provoke deficiencies in DNA damage repair, which synergizes with chemotherapy and PARP inhibitors [30]. Thus, CDK12/CDK13 inhibitors may be a promising treatment option for breast cancer.

In this report, the expression of CDK9/10/11/12/19/20 was not significantly correlated with the prognosis of patients with breast cancer. Among those CDKs, the expression levels of CDK10 and CDK19 were significantly higher in breast cancer than in normal breast tissues in Oncomine and the HPA datasets. Meanwhile, mRNA expression levels of CDK9/12/20 were significantly higher in breast cancer than in normal breast tissues in the Oncomine database, but did not differ at the protein level in the HPA. The novel CDK9 inhibitor MC180295 was reported to possess broad antitumor efficacy *in vitro* and *in vivo*, and to sensitize to immune checkpoint inhibition *in vivo* [31]. Several clinical trials evaluating CDK9 inhibitors in advanced malignancies are currently ongoing. CDK10 was identified as a determinant of endocrine therapy resistance in breast cancer, with early recurrence being observed on tamoxifen-treated ERα-positive breast cancer patients with low CDK10 expression levels [32]. Human CDK11 is encoded by two highly homologous genes, CDK11A and CDK11B. Silencing of CDK11 expression led to significant growth inhibition and apoptosis in breast cancer cells [33]. CDK19 is a paralog of CDK8 and was identified as a therapeutic target in prostate cancer and leukemia [23, 34]. CDK20 inhibition was found to enhance the efficacy of immune-checkpoint blockade in hepatocellular carcinoma [35]. Despite all this evidence supporting important contributions of TA-CDKs to tumorigenesis, additional work is required to determine their precise role in breast cancer.

In conclusion, our results demonstrated that CDK7/10/13/19 expression is significantly higher in breast cancer than in normal breast tissues, both at the mRNA and protein levels. At the mRNA level, overexpression of CDK7 or CDK8 was associated with inferior prognosis, whereas higher CDK13 expression was associated with favorable prognosis in breast cancer patients. Furthermore, a high genetic alteration rate (56%) for TA-CDKs was observed in association with shorter OS in breast cancer patients. Our results suggest that CDK7/8/13 could be prognostic biomarkers for breast cancer patients and may offer valuable insights for the development of therapies targeting TA-CDKs in breast cancer.

The present study has several potential limitations, including the use of online data sources, the absence of validation cohorts, and the lack of mechanistic studies. Nevertheless, to our knowledge our study is the first to examine multiple cancer-related databases to address the prognostic value of TA-CDKs in breast cancer. Actually, several transcription-associated CDK inhibitors, including CDK7i (CT7001, SY-1365, SY-5609 and LY3405105), CDK8/19i (SEL120), and CDK9i (Alvocidib, TP-1287, BAY1251152 and AZD4573) have now progressed to Phase I/II clinical trials in various cancer types [8]. Still, further studies are needed to elucidate the specific mechanisms by which TA-CDKs impact breast cancer development and outcome.

## MATERIALS AND METHODS

### Oncomine database analysis

Gene expression array datasets were retrieved from Oncomine (https://www.oncomine.org), an online cancer microarray database that facilitates discovery from genome-wide expression analyses. Oncomine was used to analyze the transcription levels of TA-CDKs in different cancer tissues and corresponding adjacent normal control samples. The thresholds were restricted as follows: P-value, 0.05; fold change, 1.5; gene rank, 10%; data type, mRNA.

### GEPIA2 dataset analysis

Gene Expression Profiling Interactive Analysis (GEPIA; http://gepia2.cancer-pku.cn) is an interactive web server for analyzing RNA sequencing expression data of 9,736 tumors and 8,587 normal samples from The Cancer Genome Atlas (TCGA) and the Genotype Tissue Expression (GTEx) projects, using a standard processing pipeline. GEPIA2 provides customizable functions such as tumor/normal differential expression analysis, profiling according to cancer types or pathological stages, patient survival analysis, similar gene detection, correlation analysis, and dimensionality reduction analysis [36].

### The human protein atlas analysis

The Human Protein Atlas (HPA, https://www.proteinatlas.org) is a website that aims to map all the human proteins in cells, tissues and organs using integration of various omics technologies, including antibody-based imaging, mass spectrometry-based proteomics, transcriptomics, and systems biology. The HPA contains immunohistochemistry-based expression data for 17 main cancer types and 44 different tissue types [37]. In this study, direct comparison of protein expression of different TA-CDKs between human normal breast and breast cancer tissues was performed by analysis of immunohistochemistry data.

### UALCAN analysis

UALCAN (http://ualcan.path.uab.edu) is a comprehensive interactive web resource that provides access to publicly available cancer OMICS data (TCGA and MET500). It can be used to analyze gene expression and patient survival information based on gene expression [38]. UALCAN was used to analyze patient survival based on 10 TA-CDKs stratified by mRNA expression levels in primary breast invasive carcinoma.

### Kaplan-Meier plotter’s prognostic evaluation

The Kaplan Meier plotter (http://kmplot.com/analysis/) contains information on the effect of 54,000 genes on survival outcomes for 21 cancer types, discriminated by number at risk cases, HRs, 95% Cis, and log-rank p-values [39]. This database was used to assess the prognostic value of the mRNA expression of distinct TA-CDKs in breast cancer. To analyze relapse-free survival (RFS) and overall survival (OS) of patients with breast cancer, patients were divided into two groups by median expression (high versus low expression) and assessed by a Kaplan-Meier survival curve.

### TCGA portal analysis

The TCGA portal (http://tcgaportal.org/) is a user-friendly interactive web resource that provides access to the TCGA database. It provides key functions, including patient survival analysis by high and low gene expression, differential expression analysis by tumor subtypes, as well as expression and methylation, expression and mutation, and pan-cancer analyses [40]. The TCGA portal was accessed to perform analysis of survival stratified by mRNA expression, differential expression analysis by molecular subtypes, and expression and mutation analysis of 10 TA-CDKs.

### cBioPortal analysis

The cBioPortal for cancer genomics (http://www.cbioportal.org/) is an online open-access website for exploring, visualizing, and analyzing multidimensional cancer genomics data in the TCGA database [41]. A total of 1084 breast invasive carcinoma samples (TCGA, PanCancer Atlas) were analyzed. The genomic profiles of 10 TA-CDKs were investigated based on mutations, putative copy-number alterations from the Genomic Identification of Significant Targets in Cancer (GISTIC) tool, and mRNA expression z-scores (RNA Seq V2 RSEM). Genetic mutations in TA-CDKs and their association with OS and DFS were displayed as Kaplan-Meier curves.

### DAVID 6.8 analysis

The Database for Annotation, Visualization, and Integrated Discovery (DAVID) v6.8 (https://david.ncifcrf.gov) is a comprehensive, functional annotation web-accessible tool that allows investigating the biological function of submitted genes [42]. DAVID 6.8 was used to conduct Gene Ontology (GO) and Kyoto Encyclopedia of Genes and Genomes (KEGG) pathway enrichment analyses of TA-CDKs and their closely related genes.

### Metascape analysis

Metascape (http://metascape.org) is an online tool for gene annotation and interactome and enrichment analysis, facilitating also comparative analysis of datasets across multiple independent and orthogonal experiments [43]. Metascape was used to identify pathways and to perform enrichment analysis of transcription-associated CDKs and their closely related genes. Only terms with minimum overlap of 3, P-value < 0.05, and minimum enrichment of 3 were included. The identified enriched ontology clusters were converted into a network layout. Protein-protein interaction enrichment analysis was carried out on the BioGrid, InWeb_IM, and OmniPath databases. The MCODE algorithm was then applied to the protein–protein interaction network to identify modules with densely connected proteins.

### Statistical analysis

Survival was estimated by the Kaplan–Meier method, and differences were compared by the log-rank test. P < 0.05 indicated a significant difference.

### Availability of data and materials

The datasets analyzed on this study can be found in the Oncomine, GEPIA2, the Human Protein Atlas, UALCAN, the Kaplan-Meier Plotter, TCGA portal, and cBioPortal web resources; requests for further access to datasets can be directed to yanglu@sysucc.org.cn.

### Ethics approval and consent to participate

This study was approved by the Medical Ethics Committee of Sun Yat-Sen University Cancer Center and conducted according to the principles expressed in the Declaration of Helsinki. All the datasets were retrieved from published studies and online databases, therefore informed consent from participants in the original studies has been granted.

## Supplementary Material

Supplementary Figures

## References

[1] Bray F, Ferlay J, Soerjomataram I, Siegel RL, Torre LA, Jemal A. Global cancer statistics 2018: GLOBOCAN estimates of incidence and mortality worldwide for 36 cancers in 185 countries. CA Cancer J Clin. 2018; 68:394–424. 10.3322/caac.21492 30207593

[2] Harbeck N, Gnant M. Breast cancer. Lancet. 2017; 389:1134–50. 10.1016/S0140-6736(16)31891-8 27865536

[3] Bianchini G, Balko JM, Mayer IA, Sanders ME, Gianni L. Triple-negative breast cancer: challenges and opportunities of a heterogeneous disease. Nat Rev Clin Oncol. 2016; 13:674–90. 10.1038/nrclinonc.2016.66 27184417 PMC5461122

[4] Kennecke H, Yerushalmi R, Woods R, Cheang MC, Voduc D, Speers CH, Nielsen TO, Gelmon K. Metastatic behavior of breast cancer subtypes. J Clin Oncol. 2010; 28:3271–77. 10.1200/JCO.2009.25.9820 20498394

[5] Hanahan D, Weinberg RA. Hallmarks of cancer: the next generation. Cell. 2011; 144:646–74. 10.1016/j.cell.2011.02.013 21376230

[6] Malumbres M. Cyclin-dependent kinases. Genome Biol. 2014; 15:122. 10.1186/gb4184 25180339 PMC4097832

[7] Whittaker SR, Mallinger A, Workman P, Clarke PA. Inhibitors of cyclin-dependent kinases as cancer therapeutics. Pharmacol Ther. 2017; 173:83–105. 10.1016/j.pharmthera.2017.02.008 28174091 PMC6141011

[8] Chou J, Quigley DA, Robinson TM, Feng FY, Ashworth A. Transcription-associated cyclin-dependent kinases as targets and biomarkers for cancer therapy. Cancer Discov. 2020; 10:351–70. 10.1158/2159-8290.CD-19-0528 32071145

[9] Goel S, DeCristo MJ, McAllister SS, Zhao JJ. CDK4/6 inhibition in cancer: beyond cell cycle arrest. Trends Cell Biol. 2018; 28:911–25. 10.1016/j.tcb.2018.07.002 30061045 PMC6689321

[10] O’Leary B, Finn RS, Turner NC. Treating cancer with selective CDK4/6 inhibitors. Nat Rev Clin Oncol. 2016; 13:417–30. 10.1038/nrclinonc.2016.26 27030077

[11] Spangler L, Wang X, Conaway JW, Conaway RC, Dvir A. TFIIH action in transcription initiation and promoter escape requires distinct regions of downstream promoter DNA. Proc Natl Acad Sci USA. 2001; 98:5544–49. 10.1073/pnas.101004498 11331764 PMC33249

[12] Patel H, Abduljabbar R, Lai CF, Periyasamy M, Harrod A, Gemma C, Steel JH, Patel N, Busonero C, Jerjees D, Remenyi J, Smith S, Gomm JJ, et al. Expression of CDK7, cyclin H, and MAT1 is elevated in breast cancer and is prognostic in estrogen receptor-positive breast cancer. Clin Cancer Res. 2016; 22:5929–38. 10.1158/1078-0432.CCR-15-1104 27301701 PMC5293170

[13] Ganuza M, Sáiz-Ladera C, Cañamero M, Gómez G, Schneider R, Blasco MA, Pisano D, Paramio JM, Santamaría D, Barbacid M. Genetic inactivation of Cdk7 leads to cell cycle arrest and induces premature aging due to adult stem cell exhaustion. EMBO J. 2012; 31:2498–510. 10.1038/emboj.2012.94 22505032 PMC3365431

[14] Zhang H, Christensen CL, Dries R, Oser MG, Deng J, Diskin B, Li F, Pan Y, Zhang X, Yin Y, Papadopoulos E, Pyon V, Thakurdin C, et al. CDK7 inhibition potentiates genome instability triggering anti-tumor immunity in small cell lung cancer. Cancer Cell. 2020; 37:37–54.e9. 10.1016/j.ccell.2019.11.003 31883968 PMC7277075

[15] Zhang J, Liu W, Zou C, Zhao Z, Lai Y, Shi Z, Xie X, Huang G, Wang Y, Zhang X, Fan Z, Su Q, Yin J, Shen J. Targeting super-enhancer-associated oncogenes in osteosarcoma with THZ2, a covalent CDK7 inhibitor. Clin Cancer Res. 2020; 26:2681–92. 10.1158/1078-0432.CCR-19-1418 31937612

[16] Kwiatkowski N, Zhang T, Rahl PB, Abraham BJ, Reddy J, Ficarro SB, Dastur A, Amzallag A, Ramaswamy S, Tesar B, Jenkins CE, Hannett NM, McMillin D, et al. Targeting transcription regulation in cancer with a covalent CDK7 inhibitor. Nature. 2014; 511:616–20. 10.1038/nature13393 25043025 PMC4244910

[17] Chipumuro E, Marco E, Christensen CL, Kwiatkowski N, Zhang T, Hatheway CM, Abraham BJ, Sharma B, Yeung C, Altabef A, Perez-Atayde A, Wong KK, Yuan GC, et al. CDK7 inhibition suppresses super-enhancer-linked oncogenic transcription in MYCN-driven cancer. Cell. 2014; 159:1126–39. 10.1016/j.cell.2014.10.024 25416950 PMC4243043

[18] Wang Y, Zhang T, Kwiatkowski N, Abraham BJ, Lee TI, Xie S, Yuzugullu H, Von T, Li H, Lin Z, Stover DG, Lim E, Wang ZC, et al. CDK7-dependent transcriptional addiction in triple-negative breast cancer. Cell. 2015; 163:174–86. 10.1016/j.cell.2015.08.063 26406377 PMC4583659

[19] Firestein R, Bass AJ, Kim SY, Dunn IF, Silver SJ, Guney I, Freed E, Ligon AH, Vena N, Ogino S, Chheda MG, Tamayo P, Finn S, et al. CDK8 is a colorectal cancer oncogene that regulates beta-catenin activity. Nature. 2008; 455:547–51. 10.1038/nature07179 18794900 PMC2587138

[20] Kapoor A, Goldberg MS, Cumberland LK, Ratnakumar K, Segura MF, Emanuel PO, Menendez S, Vardabasso C, Leroy G, Vidal CI, Polsky D, Osman I, Garcia BA, et al. The histone variant macroH2A suppresses melanoma progression through regulation of CDK8. Nature. 2010; 468:1105–09. 10.1038/nature09590 21179167 PMC3057940

[21] McCleland ML, Soukup TM, Liu SD, Esensten JH, de Sousa e Melo F, Yaylaoglu M, Warming S, Roose-Girma M, Firestein R. Cdk8 deletion in the Apc(Min) murine tumour model represses EZH2 activity and accelerates tumourigenesis. J Pathol. 2015; 237:508–19. 10.1002/path.4596 26235356

[22] Gu W, Wang C, Li W, Hsu FN, Tian L, Zhou J, Yuan C, Xie XJ, Jiang T, Addya S, Tai Y, Kong B, Ji JY. Tumor-suppressive effects of CDK8 in endometrial cancer cells. Cell Cycle. 2013; 12:987–99. 10.4161/cc.24003 23454913 PMC3637357

[23] Pelish HE, Liau BB, Nitulescu II, Tangpeerachaikul A, Poss ZC, Da Silva DH, Caruso BT, Arefolov A, Fadeyi O, Christie AL, Du K, Banka D, Schneider EV, et al. Mediator kinase inhibition further activates super-enhancer-associated genes in AML. Nature. 2015; 526:273–76. 10.1038/nature14904 26416749 PMC4641525

[24] Koehler MF, Bergeron P, Blackwood EM, Bowman K, Clark KR, Firestein R, Kiefer JR, Maskos K, McCleland ML, Orren L, Salphati L, Schmidt S, Schneider EV, et al. Development of a potent, specific CDK8 kinase inhibitor which phenocopies CDK8/19 knockout cells. ACS Med Chem Lett. 2016; 7:223–28. 10.1021/acsmedchemlett.5b00278 26985305 PMC4789660

[25] McDermott MS, Chumanevich AA, Lim CU, Liang J, Chen M, Altilia S, Oliver D, Rae JM, Shtutman M, Kiaris H, Győrffy B, Roninson IB, Broude EV. Inhibition of CDK8 mediator kinase suppresses estrogen dependent transcription and the growth of estrogen receptor positive breast cancer. Oncotarget. 2017; 8:12558–75. 10.18632/oncotarget.14894 28147342 PMC5355036

[26] Wu YM, Cieślik M, Lonigro RJ, Vats P, Reimers MA, Cao X, Ning Y, Wang L, Kunju LP, de Sarkar N, Heath EI, Chou J, Feng FY, et al, and PCF/SU2C International Prostate Cancer Dream Team. Inactivation of CDK12 delineates a distinct immunogenic class of advanced prostate cancer. Cell. 2018; 173:1770–82.e14. 10.1016/j.cell.2018.04.034 29906450 PMC6084431

[27] Bayles I, Krajewska M, Pontius WD, Saiakhova A, Morrow JJ, Bartels C, Lu J, Faber ZJ, Fedorov Y, Hong ES, Karnuta JM, Rubin B, Adams DJ, et al. Ex vivo screen identifies CDK12 as a metastatic vulnerability in osteosarcoma. J Clin Invest. 2019; 129:4377–92. 10.1172/JCI127718 31498151 PMC6763270

[28] Zeng M, Kwiatkowski NP, Zhang T, Nabet B, Xu M, Liang Y, Quan C, Wang J, Hao M, Palakurthi S, Zhou S, Zeng Q, Kirschmeier PT, et al. Targeting MYC dependency in ovarian cancer through inhibition of CDK7 and CDK12/13. Elife. 2018; 7:e39030. 10.7554/eLife.39030 30422115 PMC6251623

[29] Choi HJ, Jin S, Cho H, Won HY, An HW, Jeong GY, Park YU, Kim HY, Park MK, Son T, Min KW, Jang KS, Oh YH, et al. CDK12 drives breast tumor initiation and trastuzumab resistance via Wnt and IRS1-ErbB-PI3K signaling. EMBO Rep. 2019; 20:e48058. 10.15252/embr.201948058 31468695 PMC6776914

[30] Quereda V, Bayle S, Vena F, Frydman SM, Monastyrskyi A, Roush WR, Duckett DR. Therapeutic targeting of CDK12/CDK13 in triple-negative breast cancer. Cancer Cell. 2019; 36:545–58.e7. 10.1016/j.ccell.2019.09.004 31668947

[31] Zhang H, Pandey S, Travers M, Sun H, Morton G, Madzo J, Chung W, Khowsathit J, Perez-Leal O, Barrero CA, Merali C, Okamoto Y, Sato T, et al. Targeting CDK9 reactivates epigenetically silenced genes in cancer. Cell. 2018; 175:1244–58.e26. 10.1016/j.cell.2018.09.051 30454645 PMC6247954

[32] Iorns E, Turner NC, Elliott R, Syed N, Garrone O, Gasco M, Tutt AN, Crook T, Lord CJ, Ashworth A. Identification of CDK10 as an important determinant of resistance to endocrine therapy for breast cancer. Cancer Cell. 2008; 13:91–104. 10.1016/j.ccr.2008.01.001 18242510

[33] Kren BT, Unger GM, Abedin MJ, Vogel RI, Henzler CM, Ahmed K, Trembley JH. Preclinical evaluation of cyclin dependent kinase 11 and casein kinase 2 survival kinases as RNA interference targets for triple negative breast cancer therapy. Breast Cancer Res. 2015; 17:19. 10.1186/s13058-015-0524-0 25837326 PMC4344788

[34] Brägelmann J, Klümper N, Offermann A, von Mässenhausen A, Böhm D, Deng M, Queisser A, Sanders C, Syring I, Merseburger AS, Vogel W, Sievers E, Vlasic I, et al. Pan-cancer analysis of the mediator complex transcriptome identifies CDK19 and CDK8 as therapeutic targets in advanced prostate cancer. Clin Cancer Res. 2017; 23:1829–40. 10.1158/1078-0432.CCR-16-0094 27678455

[35] Zhou J, Liu M, Sun H, Feng Y, Xu L, Chan AW, Tong JH, Wong J, Chong CC, Lai PB, Wang HK, Tsang SW, Goodwin T, et al. Hepatoma-intrinsic CCRK inhibition diminishes myeloid-derived suppressor cell immunosuppression and enhances immune-checkpoint blockade efficacy. Gut. 2018; 67:931–44. 10.1136/gutjnl-2017-314032 28939663 PMC5961939

[36] Tang Z, Kang B, Li C, Chen T, Zhang Z. GEPIA2: an enhanced web server for large-scale expression profiling and interactive analysis. Nucleic Acids Res. 2019; 47:W556–60. 10.1093/nar/gkz430 31114875 PMC6602440

[37] Uhlen M, Zhang C, Lee S, Sjöstedt E, Fagerberg L, Bidkhori G, Benfeitas R, Arif M, Liu Z, Edfors F, Sanli K, von Feilitzen K, Oksvold P, et al. A pathology atlas of the human cancer transcriptome. Science. 2017; 357:eaan2507. 10.1126/science.aan2507 28818916

[38] Chandrashekar DS, Bashel B, Balasubramanya SA, Creighton CJ, Ponce-Rodriguez I, Chakravarthi BV, Varambally S. UALCAN: a portal for facilitating tumor subgroup gene expression and survival analyses. Neoplasia. 2017; 19:649–58. 10.1016/j.neo.2017.05.002 28732212 PMC5516091

[39] Györffy B, Lanczky A, Eklund AC, Denkert C, Budczies J, Li Q, Szallasi Z. An online survival analysis tool to rapidly assess the effect of 22,277 genes on breast cancer prognosis using microarray data of 1,809 patients. Breast Cancer Res Treat. 2010; 123:725–31. 10.1007/s10549-009-0674-9 20020197

[40] Xu S, Feng Y, Zhao S. Proteins with evolutionarily hypervariable domains are associated with immune response and better survival of basal-like breast cancer patients. Comput Struct Biotechnol J. 2019; 17:430–40. 10.1016/j.csbj.2019.03.008 30996822 PMC6451114

[41] Cerami E, Gao J, Dogrusoz U, Gross BE, Sumer SO, Aksoy BA, Jacobsen A, Byrne CJ, Heuer ML, Larsson E, Antipin Y, Reva B, Goldberg AP, et al. The cBio cancer genomics portal: an open platform for exploring multidimensional cancer genomics data. Cancer Discov. 2012; 2:401–04. 10.1158/2159-8290.CD-12-0095 22588877 PMC3956037

[42] Huang DW, Sherman BT, Lempicki RA. Systematic and integrative analysis of large gene lists using DAVID bioinformatics resources. Nat Protoc. 2009; 4:44–57. 10.1038/nprot.2008.211 19131956

[43] Zhou Y, Zhou B, Pache L, Chang M, Khodabakhshi AH, Tanaseichuk O, Benner C, Chanda SK. Metascape provides a biologist-oriented resource for the analysis of systems-level datasets. Nat Commun. 2019; 10:1523. 10.1038/s41467-019-09234-6 30944313 PMC6447622

