# Supplementary material for: Expression and prognostic value of transcription-associated cyclin-dependent kinases in human breast cancer
Source: Aging (Albany NY). 2021 Mar 3;13(6):8095–114. doi: 10.18632/aging.202595 (PMC8034920; doi:10.18632/aging.202595)
Supplement: Supplementary Figures [file aging-13-202595-s001.pdf]

SUPPLEMENTARY FIGURES

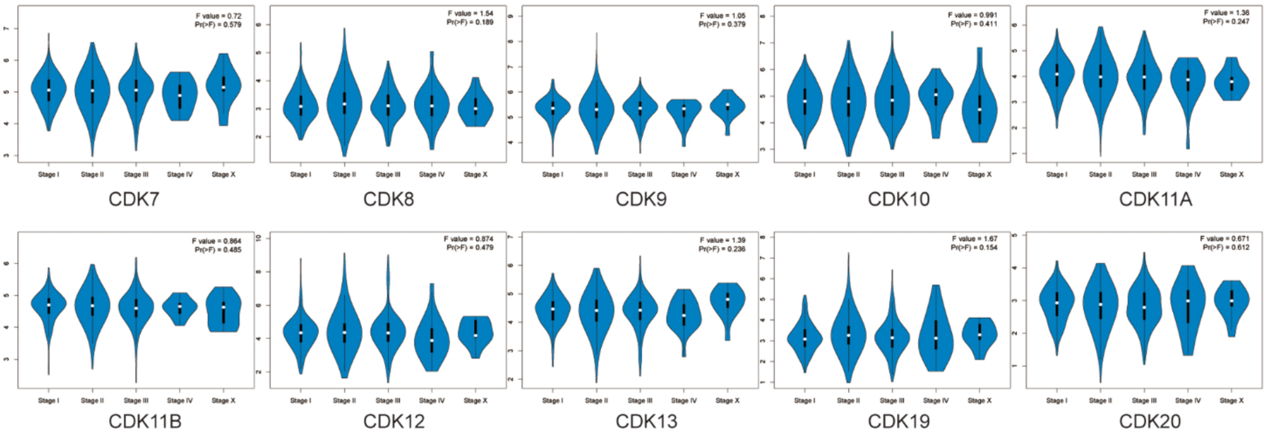

Supplementary Figure 1. Correlation between transcription-associated CDKs expression and tumor stage in breast cancer patients (GEPIA2).

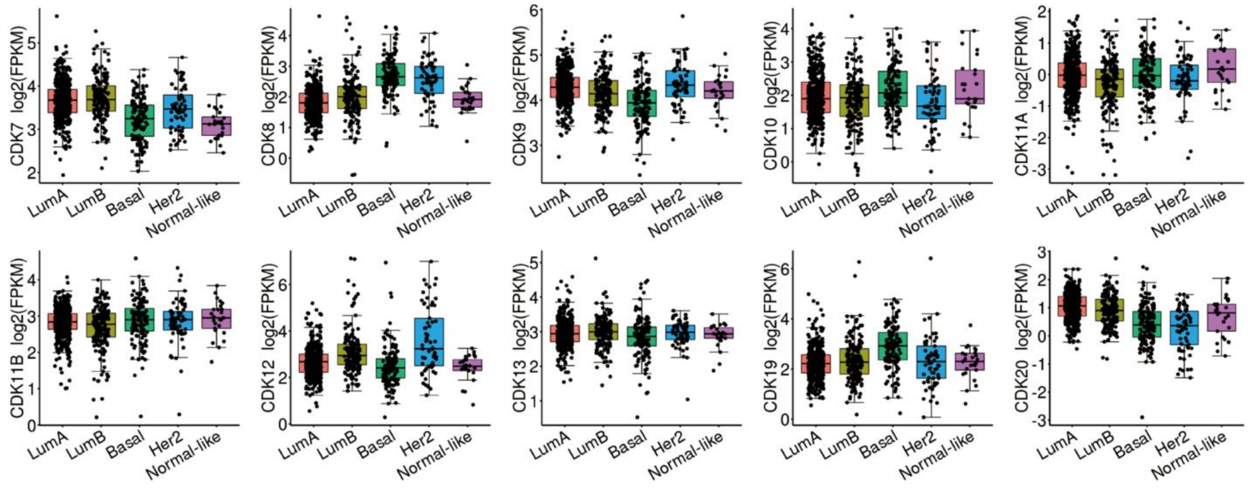

Supplementary Figure 2. Correlation between transcription-associated CDKs expression and molecular subtype in breast cancer patients (TCGA portal).

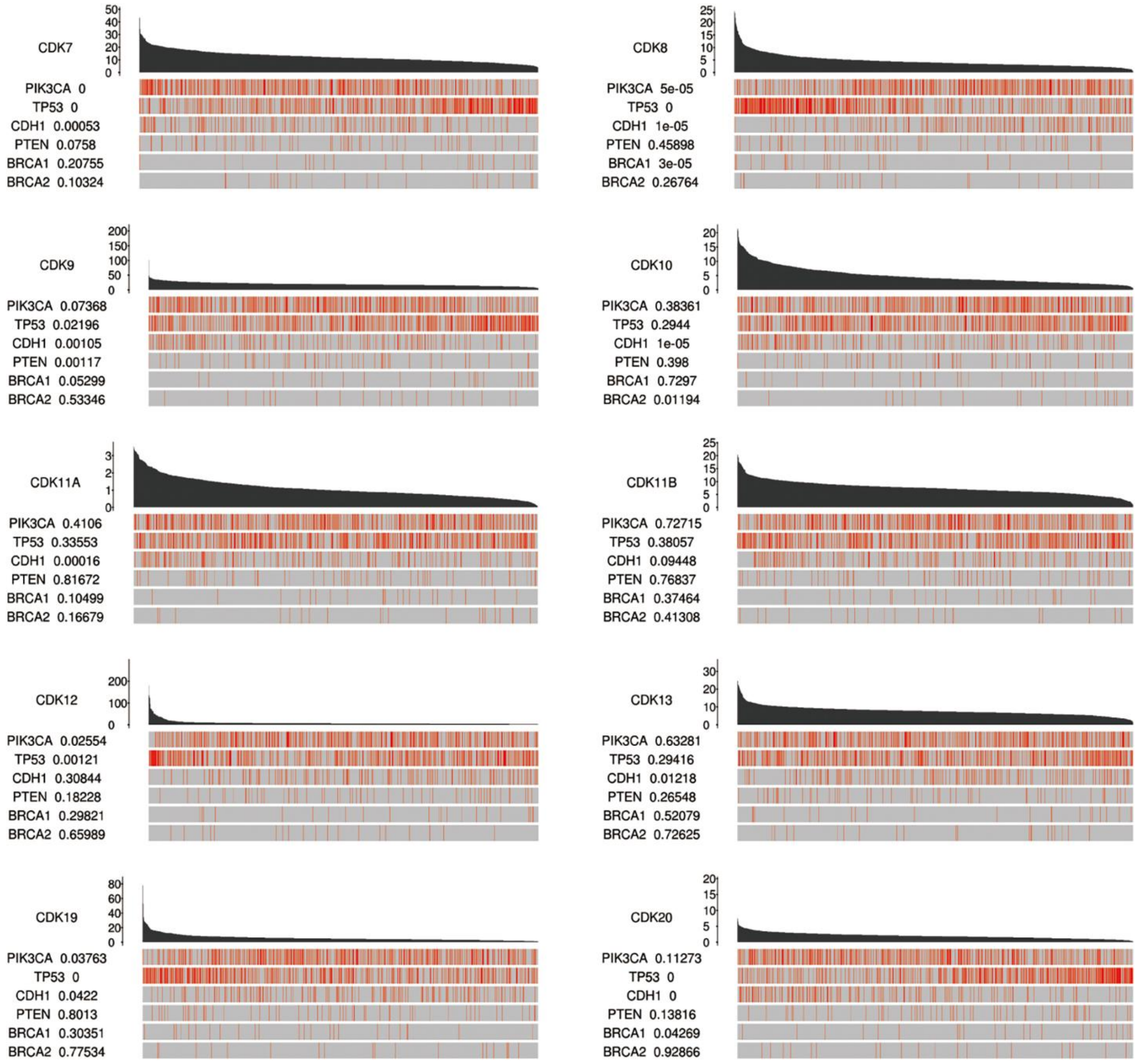

Supplementary Figure 3. Correlation between the expression of transcription-associated CDKs and the mutation of highly mutated genes in breast cancer patients (TCGA portal). The driver mutated (red) and not-mutated (gray) samples are shown.

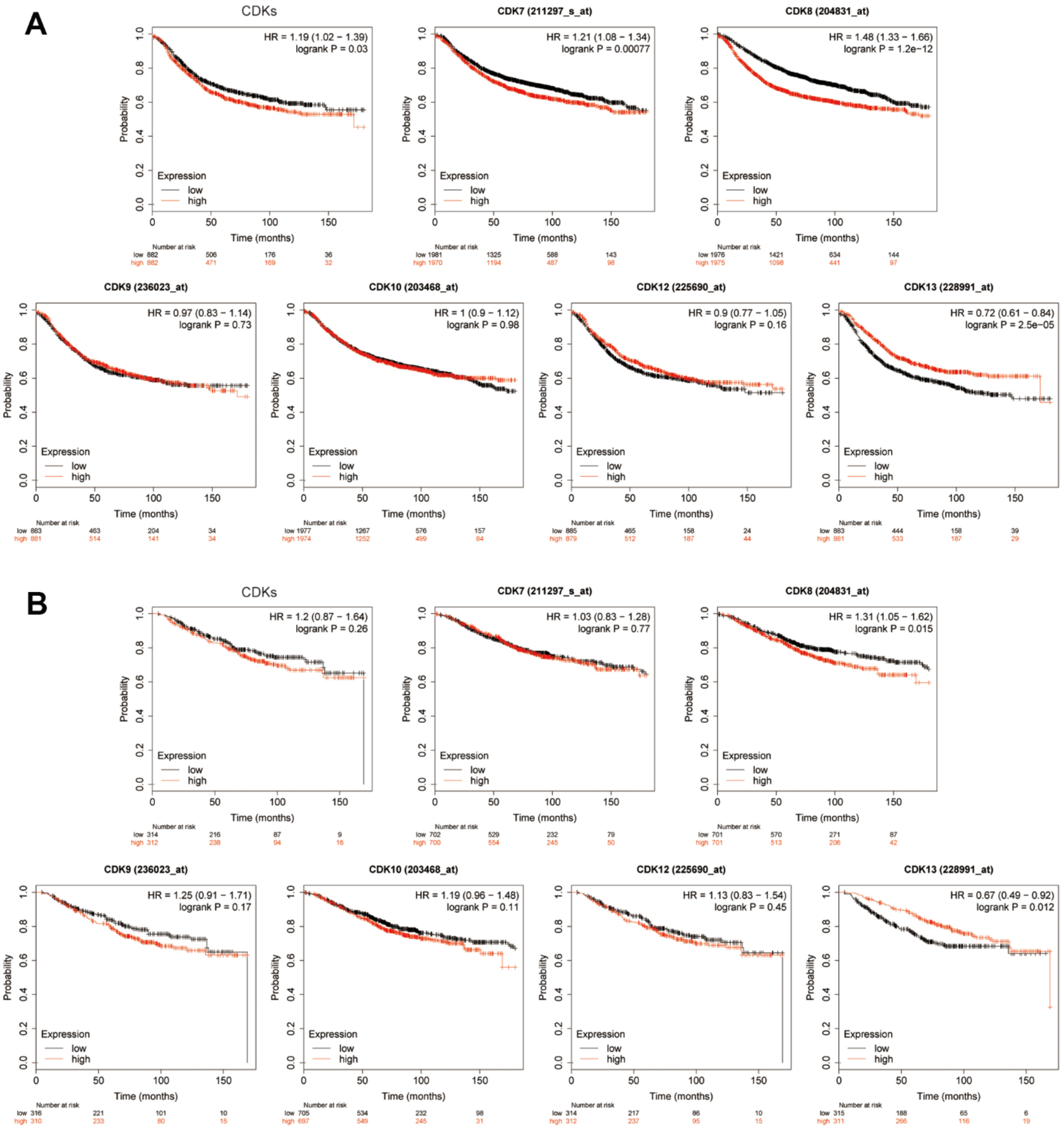

Supplementary Figure 4. Prognostic value of mRNA expression of distinct transcription-associated CDKs in breast cancer patients by Kaplan-Meier Plotter tool. (**A**) Relapse-free survival; (**B**) Overall survival.
